# Efficacy and safety of a new drug formulation, amoxicillin-clavulanate-cineole, for adult lower respiratory tract infections: a nationwide observational study in Morocco

**DOI:** 10.3389/fphar.2025.1549014

**Published:** 2025-05-30

**Authors:** Mohamed Chakib Benjelloun, Youness El Achhab, Chakib Nejjari, the ORCA Study Group

**Affiliations:** ^1^ Faculty of Medicine, Pharmacy and Dentistry, Sidi Mohammed Ben Abdellah University, Fez, Morocco; ^2^ Euromed Research Center, Euromed University of Fes, Fez, Morocco; ^3^ Department of Biology, Regional Center of Education and Training Professions of Fes-Meknes, Fez, Morocco

**Keywords:** lower respiratory tract infections, community, efficacy, safety, Olipen^®^

## Abstract

**Background:**

Lower respiratory tract infections (LRTIs) remain significant global health threats, causing substantial morbidity and mortality. The treatment landscape for LRTIs has evolved significantly, presenting increasing challenges due to rising antibiotic resistance and frequent treatment failures. This study aims to examine the real-life efficacy and safety of a new drug formulation, Olipen^®^ (amoxicillin-clavulanate-cineole), in adult patients with LRTIs within the community setting.

**Methods:**

This observational, non-interventional study recruited 936 patients. Olipen^®^ 500 mg (amoxicillin 500 mg, clavulanate 62.5 mg, cineole 100 mg) was administered as two sachets, three times daily, for 7–14 days, as per clinical practice guidelines. The primary outcome focused on the clinical recovery and safety as a secondary outcome.

**Results:**

A total of 936 patients were enrolled in the study at the national level. Nearly all patients (94.9%) achieved clinical recovery. Therapeutic failure was reported in 26 patients (2.8%), while the outcome remained undetermined for 22 patients (2.3%). After 3–4 days of treatment, 57.8% of patients were symptom-free. Radiologically, 81% of patients showed improvement during follow-up. Treatment effectiveness is not affected patient characteristics, whereas chronic cough and dyspnea may hinder clinical recovery in pa-tients with LTRIs. Olipen^®^ was well tolerated, with most of the adverse events reported were considered non-serious and most of them were resolved (87.5%).

**Conclusion:**

Olipen^®^ was found to be effective and well tolerated in adults with acute exacerbation of chronic bronchitis/COPD, community-acquired pneumonia or superinfection as well as adult patients with pathological lung.

## 1 Introduction

Lower respiratory tract infections (LRTIs) remain a significant global health concern, contributing to substantial morbidity and mortality (Feldman and Shaddock, 2019). These infections include a spectrum of conditions such as influenza-like illnesses, acute and chronic bronchitis (including exacerbations), and pneumonia. LRTIs are typically acute illnesses lasting up to 21 days, primarily presenting with cough and often accompanied by other symptoms such as sputum production, dyspnea, wheezing, or chest discomfort/pain (Woodhead et al., 2011).

The incidence of LRTIs is strongly influenced by risk factors. Age is a major determinant, as aging is associated with gradual impairments in immune defenses, often exacerbated by comorbidities (Feldman and Shaddock, 2019). Environmental and socioeconomic conditions also significantly contribute to infection risk, as shown in studies across both developed and developing regions (Chen et al., 2014; Feldman and Shaddock, 2019). While viral infections can predispose individuals to bacterial superinfections, the specific pathogens involved depend on multiple variables; such as age over 65 years, corticosteroid use, cardiac disease, seasonality, structural lung disease, and recent antibiotic use (Hossain et al., 2019; Unger and Bogaert, 2017; Wilson, 2006). A deeper understanding of these interactions can guide the development of novel therapies and improve prevention strategies by better identifying high-risk populations.

Antibiotic selection should prioritize rapid bactericidal activity with minimal side effects; namely, toxicity, drug interactions, and resistance development. Based on these principles, beta-lactam antibiotics such as amoxicillin and its combination with clavulanic acid are recommended as first-line oral treatments for mild to moderate and some severe LRTIs (Cantón et al., 2022; Huttner et al., 2020; Mahashur, 2018; Malakounidou et al., 2023; White et al., 2004; Woodhead et al., 2011). Amoxicillin–clavulanate, in clinical use for over 4 decades, offers broad-spectrum activity, including coverage of β-lactamase-producing organisms. Both components have good oral bioavailability and lack significant pharmacokinetic interactions (Sánchez Navarro, 2005; Ball, 2007; File, 2007). The combination’s safety profile is well-established, with gastrointestinal symptoms (especially diarrhea) being the most commonly reported adverse events. These are usually mild, transient, and self-limiting (Salvo et al., 2009).

Despite appropriate antibiotic therapy, some patients with LRTIs fail to achieve full recovery, highlighting the need for adjunctive treatments (Cazzola et al., 2017). Although systemic corticosteroids may benefit certain cases of pneumonia, particularly severe community-acquired pneumonia, their use remains controversial (Cazzola et al., 2017; Liapikou and Torres, 2016).

Emerging evidence supports the therapeutic potential of 1,8-cineole, a monoterpene with anti-inflammatory and mucolytic effects. Its activity in chronic respiratory conditions has been linked to modulation of inflammatory pathways and reduction of mucus hypersecretion (Pries et al., 2023; Sudhoff et al., 2015). Notably, combining amoxicillin with 1,8-cineole has been shown to reduce the antibiotic’s affinity for β-lactamase, thereby enhancing its efficacy against ESBL-producing bacteria (Akhmouch et al., 2022a). Including a β-lactamase inhibitor such as clavulanic acid may further potentiate this effect (Remmal and Akhmouch, 2019).

Widely used in phytotherapy, 1,8-cineole exhibits broad-spectrum antimicrobial, mucolytic, and anti-inflammatory properties, with potential antiviral activity also being explored (Cai et al., 2021; Pries et al., 2023). Clinical studies have confirmed its favorable safety profile. A phase III trial by Kardos et al. (2021) reported a low incidence of mild, non-serious adverse events among patients with acute bronchitis.

Combining antibiotics with plant-derived bioactives such as cineole may usher in a new era of phytopharmaceuticals, offering strategies to combat multidrug-resistant pathogens and reduce reliance on conventional antibiotics (Akhmouch et al., 2022a; Hriouech et al., 2020; Kwiatkowski et al., 2020).

In 2021, Moroccan authorities approved Olipen^®^, a novel formulation combining amoxicillin, clavulanic acid, and cineole. To date no registered human trial has examined the fixed combination of amoxicillin–clavulanate with cineole. Nevertheless, pre-clinical studies show that adding 1,8-cineole to amoxicillin-clavulanate markedly enhances amoxicillin bioavailability and suppresses β-lactamase-mediated resistance (Akhmouch et al., 2022a; Akhmouch et al., 2022b). Cineole alone has proven clinical benefit in airway infections: double-blind RCTs demonstrated faster symptom resolution in acute bronchitis (Fischer and Dethlefsen, 2013), fewer exacerbations in COPD (Worth et al., 2009), and reduced steroid requirements in asthma (Juergens et al., 2003). These data provided the biological rationale for evaluating the fixed Olipen^®^ formulation in real-world LRTIs.

This observational study aims to evaluate the safety and effectiveness of Olipen^®^ in adult outpatients with LRTIs, based on real-world data reflecting current clinical practice.

## 2 Materials and methods

### 2.1 Study design and participants

The clinical research protocol was reviewed and approved by the Faculty of Medicine and Pharmacy of Casablanca Research Ethics Committee. All research procedures complied with the principles outlined in the Declaration of Helsinki. Informed consent was obtained from all patients.

Recruitment was nationwide, encompassing all 12 administrative regions of Morocco. Consecutive adult out-patients were enrolled in three categories of sites: (i) the five university teaching hospitals (CHU Rabat, Casablanca, Fez, Marrakech, and Oujda), which serve as tertiary referral centers and account for most of LRTIs admissions nationally; (ii) eight high-volume regional or provincial public hospitals, selected to capture semi-urban and rural catchment areas; and (iii) 42 private pulmonology clinics distributed across major cities and medium-sized towns. This mix of public-sector tertiary and secondary facilities together with community-based private practices was chosen to ensure (a) comprehensive geographic representation, (b) inclusion of diverse care pathways (hospital-initiated vs ambulatory treatment), and (c) feasibility of recruiting the targeted sample size within the study window.

The study spanned from the enrollment of the first patient in December 2022 to the last patient in October 2023 with a consecutive convenience sampling. For this cohort, the selection criteria were as follows: 1) outpatients aged 18 years and above; 2) the trial investigator opted for probabilistic broad-spectrum antibiotherapy; 3) clinical diagnosis of mild-to-moderate community-acquired pneumonia, acute exacerbation of chronic bronchitis/COPD, or superinfection of a pathological lung. The exclusion criteria included: 1) documented hypersensitivity to amoxicillin, clavulanate, 1,8-cineole, any other β-lactam antibiotic, or study excipients (these patients were not prescribed Olipen^®^ and were managed with an alternative antibiotic); 2) history of epilepsy or any seizure disorder, because both 1,8-cineole and β-lactam antibiotics have been reported to lower the seizure threshold and provoke seizures in susceptible individuals; and 3) pregnant or breastfeeding women.

The study was conducted over 6 months with 2 principal visits. Clinic visits occurred at inclusion and post-treatment. During the administration visit, a detailed physical examination, including thoracic radiography, was performed, and risk factors, clinical signs, and symptoms were collected. At the post-treatment visit, medication adherence, clinical efficacy, adverse events, and a brief physical examination were assessed. For pneumonia, the follow-up visit was scheduled between days 10 and 14, while for other diseases, it was scheduled between days 7 and 10.

The study was implemented nationwide across 55 Moroccan facilities under the supervision of 62 principal investigators. Before enrolment began, a certified clinical-research trainer travelled to each site to deliver on-site good clinical practice instruction and to supply a standardized case-report form (CRF) that contained tick-boxes and coded fields to harmonize data capture. Recruitment lasted 6 months and required two protocol-defined visits. At Visit 1 (inclusion) the investigator obtained written informed consent, recorded risk factors, clinical signs and symptoms, performed a comprehensive physical examination with chest radiography and initiated Olipen^®^ therapy. Visit 2 (post-treatment follow-up) occurred on Days 10–14 for community-acquired pneumonia and on Days 7–10 for other LRTIs; during this visit investigators assessed medication adherence, clinical efficacy, adverse events and conducted a focused examination. Completed CRFs were couriered weekly to the coordinating center, where double data entry and discrepancy checks ensured consistency across all sites.

### 2.2 Treatment

According to legal information (Moroccan agency for medicine and health products, 2021), Olipen^®^ 500 mg (amoxicillin 500 mg -clavulanate 62.5 mg -cineole 100 mg) is administered as 2 sachets, 3 times a day, for 7–10 days in cases of acute exacerbation of chronic bronchitis/COPD, and for 10–14 days in cases of community-acquired pneumonia or superinfection of pathological lung.

### 2.3 Outcomes

For efficacy, the treating physician performed a global clinical assessment at Visit 2 and classified each case as clinical cure (return to pre-infection status and disappearance of all signs/symptoms), clinical improvement (symptoms improving without the need for another antibiotic), or therapeutic failure (unchanged or worsened symptoms, need for an additional or alternative antibiotic, or death from LRTI complications); indeterminate outcomes are noted when post-treatment data are missing. In community-acquired pneumonia, the chest radiograph obtained at Visit 2 was compared with baseline to document radiologic resolution or improvement.

Safety monitoring relied on the systematic capture of all treatment-emergent adverse events (AEs) and serious adverse events (SAEs) in accordance with the protocol’s standard definitions. Investigators recorded each AE—assigning a severity grade and assessing its relationship to Olipen®—on the pharmacovigilance section of the paper CRF. Any SAE or predefined “alert term” was reported to the sponsor by fax within 24 h, whereas non-serious AEs were submitted with the regular CRF batches for central double data entry and reconciliation.

### 2.4 Statistical analysis

Data analysis was performed using Jamovi software (2.3.28). Descriptive analysis was performed for data exploration and were exhibited as the mean ± standard deviation (95% confidence interval of the mean) for continuous variables. Categorical variables were summarized using counts and percentages. The Mann-Whitney U test was used in continuous variables between two groups of independent samples based on whether the data were in normal distribution according to a Kolmogorov-Smirnov test. Chi-squared test was used to compare the differences between different groups.

## 3 Results

### 3.1 Demographic and disease characteristics at baseline

A total of 936 patients were enrolled in the study from 60 centers at the national level. Table 1 presents the characteristics and clinical features of the study population. The mean age was 56.3 ± 18.3 years, ranging from 18 to 96 years. Notably, patients aged 65 years and older constituted 39.2% of the cases. Chronic obstructive pulmonary disease (COPD) and/or interstitial pneumonias was the most common risk factor for LTRIs, accounting for approximately 27.4% of the cases. As antecedent clinical signs, chronic cough and chronic dyspnea were observed in 56.6% and 46.8% of the cases, respectively. On the day of the exam, cough was the most prevalent clinical sign, present in 84.2% of the cases. Use of antibiotics other than Olipen^®^ was noted in 26.9% of the cases, with half of those patients having used them within the last 10 days.

**TABLE 1:** Demographic and disease characteristics at baseline (n = 936).

Characteristics	Mean ± SD or n (%)
Age, years	56,3 ± 18,3
Age ≥65 years	367 (39.2%)
Female	472 (50.4%)
Risk factors	
Current smoker	220 (23.5%)
COPD and/or interstitial pneumonias	256 (27.4%)
Asthma	202 (21.6%)
Cardiac diseases	192 (20.5%)
Diabetes	129 (13.8%)
Antecedent clinical signs	
Chronic cough^a^	530 (56.6%)
Chronic dyspnea^b^	438 (46.8%)
mMRC dyspnea grade ≥3	154 (35.2%)
Current clinical signs	
Fever	458 (48.9%)
Thoracic pain	338 (36.1%)
Cough	785 (84.2%)
Purulent expectoration	562 (60.0%)
Dyspnea	562 (60.0%)
Abnormal chest radiography	635 (67.8%)
Antecedent use of antibiotics	252 (26.9%)
Time to last treatment less than 10 days *(n = 252)*	120 (47.6%)

COPD, chronic obstructive pulmonary disease; mMRC, modified medical research council; SD, standard deviation.

^a^ Chronic cough: cough lasting >8 weeks (CHEST, guideline: Irwin et al., 2018).

^b^ Chronic dyspepsia: upper abdominal discomfort persisting >4 weeks (Rome IV: Mounsey et al., 2020).

### 3.2 Efficacy analysis

The primary endpoint was the proportion of patients classified as clinical cure at the post treatment visit, where clinical cure was defined *a priori* as a return to the pre infection state with complete disappearance of all signs and symptoms of the LRTI. The majority of patients (64.6%) received Olipen^®^ treatment for 7–8 days. Treatment discontinuation was reported in two patients; one experienced vomiting with epigastric pain, while the other chose to stop the treatment on their own.

Nearly all patients (94.9%) achieved clinical recovery. Therapeutic failure was reported in 26 patients (2.8%), while the outcome remained undetermined for 22 patients (2.3%). After 3–4 days of treatment, 57.8% of patients were symptom-free. Radiologically, 81% of patients showed improvement during follow-up. Only 6 patients (1.1%) reported a worsening of their radiological status, and 101 patients (17.9%) exhibited no change.

Table 2 illustrates the clinical effectiveness by detailing patient characteristics. Chronic cough and dyspnea can impede clinical recovery in patients with LTRIs. Other patient characteristics were not associated with effectiveness of the Olipen^®^ treatment.

**TABLE 2:** Olipen^®^ treatment efficiency by patient characteristics (n = 936).

Characteristics		Clinical response		*p*-value
		Clinical recovery	Treatment failure or ND	
Age, years *(Mean ± SD)*		56.2 ± 18.4	58.5 ± 17.5	0.421*
Age	<65 years	535 (94.9%)	29 (5.1%)	0.981
	≥65 years	348 (94.8%)	19 (5.2%)	
Sex	Female	449 (95.1%)	23 (4.9%)	0.704
	Male	436 (94.6%)	25 (5.4%)	
Smoking	Current smoker	206 (93.6%)	14 (6.4%)	0.342
	No smoking	682 (95.3%)	34 (4.7%)	
Diabetes	Yes	118 (91.5%)	11 (8.5%)	0.059
	No	770 (95.4%)	37 (4.6%)	
Asthma	Yes	192 (95.0%)	10 (5.0%)	0.897
	No	696 (94.8%)	38 (5.2%)	
Cardiac diseases	Yes	178 (92.7%)	14 (7.3%)	0.127
	No	710 (95.4%)	34 (4.6%)	
COPD	Yes	240 (93.8%)	16 (6.3%)	0.340
	No	648 (95.3%)	32 (4.7%)	
Antecedent clinical signs				
*Chronic cough*	Yes	496 (93.6%)	34 (6.4%)	**0.041**
	No	392 (96.6%)	14 (3.4%)	
*Chronic dyspnea*	Yes	410 (93.6%)	28 (6.4%)	0.100
	No	478 (96.0%)	20 (4.0%)	
Current clinical signs				
*Fever*	Yes	438 (95.6%)	20 (4.4%)	0.301
	No	450 (94.1%)	28 (5.9%)	
*Thoracic pain*	Yes	323 (95.6%)	15 (4.4%)	0.472
	No	565 (94.5%)	33 (5.5%)	
*Cough*	Yes	743 (94.6%)	42 (5.4%)	0.523
	No	141 (95.9%)	6 (4.1%)	
*Dyspnea*	Yes	524 (93.2%)	38 (6.8%)	**0.005**
	No	364 (97.3%)	10 (2.7%)	
*Chest radiography*	Abnormal	599 (94.3%)	36 (5.7%)	0.188
	Normal	179 (96.8%)	6 (3.2%)	
Antecedent use of antibiotics	Yes	240 (95.2%)	12 (4.8%)	0.758
	No	648 (94.7%)	36 (5.3%)	

*U Mann-Whitney test.

COPD, chronic obstructive pulmonary disease; ND, not determined.

### 3.3 Safety assessments

The 62 AEs occurring in 40 patients during Olipen^®^ treatment are listed in Table 3. Gastrointestinal-related events were most common in patients. Diarrhea was the most frequent (24.2%), followed by vomiting (16.1%), nausea (14.5%), abdominal pain (9.7%) and epigastralgia (6.5%). The AEs reported in this study were considered non-serious (ICH harmonized Tripartite Guideline, 2003). They did not result in hospitalization, significant disability, surgical or medical intervention, or pose any life-threatening risks. Among 40 patients with AEs, most of the events were resolved (35 patients, 87.5%). Only three patients experienced persistent AEs up to the day of the post-treatment examination. Mild gastrointestinal disturbances were reported in 2 patients (including nausea and vomiting in one patient and diarrhea in other patient), while asthenia was reported in 1 patient. Only one patient required supportive treatment with anti-emetics, whereas the other events resolved without the need for additional medical intervention. All persistent AEs were classified as non-serious, and they resolved spontaneously within a few days. These events did not necessitate discontinuation of treatment or a change in dosage.

**TABLE 3:** Treatment-emergent adverse events in 40 patients (n = 936).

Adverse events	n (%)
Total events occurred	62 (100%)
Diarrhea	15 (24.2%)
Vomiting	10 (16.1%)
Nausea	9 (14.5%)
Abdominal pain	6 (9.7%)
Epigastralgia	4 (6.5%)
Asthenia	3 (4.8%)
Vertigo	2 (1.6%)
Thoracic pain	2 (3.2%)
Bitter taste	2 (3.2%)
Rash	1 (1.6%)
Labial Eruption	1 (1.6%)
Dyspnea	1 (1.6%)
Gastritis	1 (1.6%)
Gastric intolerance	1 (1.6%)
Fever	1 (1.6%)
Anorexia	1 (1.6%)
Urticaria	1 (1.6%)
Pruritus	1 (1.6%)

During the study, a single death was reported involving a 78-year-old male smoker who presented with a severe productive cough and dyspnea, accompanied by diffuse crepitant rales on auscultation. Radiological findings showed bronchiectasis. Despite 10 days of treatment, clinical efficacy could not be determined, and no AEs were reported for this patient. The patient had been hospitalized for pneumonia a month before his consultation with the trial investigator. The causal relationship with the product remains undetermined.

## 4 Discussion

This observational study evaluated the clinical safety and effectiveness of the newly developed Olipen^®^ formulation (a combination of amoxicillin, clavulanic acid, and cineole) in adult outpatients with LRTIs. The findings suggest that Olipen^®^ is both safe and effective for the management of mild to moderate LRTIs in a real-world setting, with the majority of patients showing significant clinical improvement and a low incidence of adverse events.

The clinical success rate observed in this study aligns with the well-established efficacy of amoxicillin–clavulanate in treating respiratory tract infections (Mahashur, 2018; Malakounidou et al., 2023). The addition of cineole appears to further enhance therapeutic outcomes, potentially through its mucolytic, anti-inflammatory, and antimicrobial properties (Akhmouch et al., 2022a; Fischer and Dethlefsen, 2013; Worth et al., 2009; Juergens et al., 2003). Preclinical studies have shown that cineole can potentiate the antibacterial activity of amoxicillin, even in the presence of β-lactamase-producing bacteria, by reducing the antibiotic’s affinity for these enzymes (Akhmouch et al., 2022a). This synergistic effect may contribute to the high rate of clinical resolution seen in our cohort.

This observational study provides real-world evidence supporting the clinical effectiveness of the enhanced pharmacokinetic formulation Olipen^®^ in adult outpatients with LTRIs. Clinical recovery was achieved in 94.9% of patients, with 57.8% becoming symptom-free after just 3–4 days of treatment, highlighting a rapid therapeutic response. These findings are consistent with previous reports on the efficacy of cineole-containing regimens in respiratory tract infections, which emphasize both anti-inflammatory and mucolytic properties that complement antibiotic action (Kardos et al., 2021). The inclusion of cineole may contribute to the enhanced symptom relief and shortened recovery time observed in this study.

In terms of radiological outcomes, 81% of patients showed improvement, reinforcing the clinical findings and suggesting resolution of inflammation and infection at the pulmonary level. Only 1.1% reported radiological worsening, and 17.9% showed no change. Therefore, it is well-documented that radiographic improvements often lag behind clinical recovery in patients with community-acquired pneumonia (Bruns et al., 2010; El Solh et al., 2004). A study by Bruns et al. (2010) observed that while 93% of patients achieved clinical cure by day 10, only 30.8% exhibited radiographic resolution at the same time point. By day 28, clinical cure was observed in 88.9% of patients, whereas radiographic resolution was noted in 68.4%. This discrepancy underscores that radiological findings may persist despite significant clinical improvement. Factors such as patient age, severity of pneumonia, and underlying comorbidities can further delay radiographic resolution. Therefore, the observation in our study that a subset of patients exhibited clinical recovery without corresponding radiological improvement aligns with established clinical patterns.

Taken together, these results suggest that Olipen^®^ may be a valuable real-world therapeutic option for outpatient management of LTRIs, especially in settings where rapid clinical response is desired. The combined antimicrobial and anti-inflammatory profile of this formulation supports its role in reducing symptom burden and potentially minimizing unnecessary use of broader-spectrum antibiotics. However, further comparative studies are warranted to establish its relative benefit over standard amoxicillin–clavulanate regimens.

Overall, amoxicillin 500 mg -clavulanate 62.5 mg -cineole 100 mg was well tolerated, with most of the AEs reported were considered non-serious and most of them were resolved (87.5%). Treatment discontinuation was noted in two patients: one due to vomiting and epigastric pain, and the other who chose to stop the treatment voluntarily. Diarrhea was the most frequently reported AE overall and also the most commonly reported AE suspected or probably related to the study medication. However, it was not the cause of study withdrawal for any patient. One death was occurred in this study and it was not considered to be related to Olipen^®^ treatment.

Diarrhea was indeed the most commonly reported adverse event (AE) in our study, consistent with the well-documented gastrointestinal side effects of amoxicillin-clavulanate combinations. The *β*-lactam class of antibiotics, particularly when combined with clavulanic acid, is known to cause diarrhea in up to 20% of patients, as reported in prior clinical trials and post-marketing surveillance (Perry and Scott, 2004; Hawkey, 1994). This is often attributed to alterations in gut microbiota and the osmotic effects of unabsorbed clavulanate. In our study, the incidence and severity of diarrhea were comparable or slightly lower than rates reported for standard amoxicillin-clavulanate, which may be due in part to the inclusion of cineole in the Olipen^®^ formulation. Cineole has demonstrated gastrointestinal-protective and anti-inflammatory properties in experimental models, potentially mitigating some of the gut-related side effects associated with antibiotic therapy (Mączka et al., 2021).

Thus, while the gastrointestinal AEs reported with Olipen^®^ are not unexpected given its antibiotic components, the addition of cineole may offer a favorable modulation of tolerability. This supports the rationale for Olipen^®^ as an optimized formulation that retains the antimicrobial efficacy of amoxicillin–clavulanate while potentially improving patient comfort and compliance.

This study offers valuable insights into the real-world use of Olipen^®^ by evaluating its clinical effectiveness, safety, and tolerability in a diverse outpatient population with LRTIs. One of the major strengths of this study is its observational design, which, although lacking the rigor of randomized controlled trials, enables the capture of routine clinical practices and patient outcomes in a naturalistic setting. This approach enhances the external validity and generalizability of the findings. The inclusion of a heterogeneous patient population, varying in disease severity, comorbidities, and geographic origin, further strengthens the relevance of the results to everyday clinical practice. Additionally, the combined use of physician-reported outcomes and patient-reported experiences offers a more holistic assessment of treatment response and tolerability. However, certain limitations must be acknowledged. The open-label nature of the study may introduce bias in outcome reporting, potentially overestimating the perceived effectiveness of treatment. Furthermore, because this was an observational study without a comparative control arm, a randomized controlled trial directly comparing Olipen^®^ with standard amoxicillin-clavulanate (or with placebo as an adjunct) would be valuable to confirm these real-world findings. Despite these limitations, the study fills an important gap by providing preliminary evidence that supports the clinical utility of Olipen^®^ in real-life outpatient care, while also laying the groundwork for future controlled studies.

In conclusion, Olipen^®^ was found to be effective and well tolerated in adults with acute exacerbation of chronic bronchitis/COPD, community-acquired pneumonia or superinfection as well as adult patients with pathological lung. Further works are also warranted to determine the efficiency of this combination taking into account viral infections.

## Data Availability

The original contributions presented in the study are included in the article/Supplementary Material, further inquiries can be directed to the corresponding author.
