# Supplementary material for: Efficacy and safety of a new drug formulation, amoxicillin-clavulanate-cineole, for adult lower respiratory tract infections: a nationwide observational study in Morocco
Source: Front Pharmacol. 2025 May 30;16:1549014. doi: 10.3389/fphar.2025.1549014 (PMC12162957; doi:10.3389/fphar.2025.1549014)
Supplement: Supplementary file 1 [file Table1.docx]

**Table S1. List of ORCA Study Investigators (ordered alphabetically by surnames)**

| **Principal Investigator** | **Institution** | **Location** |
| --- | --- | --- |
| Abdelaziz CHADLI, MD | Pulmonologist, private | Casablanca |
| Abdeljabbar BENALI, MD | Pneumo-phthisiologist & allergist, private | Khenifra |
| Abdelkarim MOUMEN, MD | Pulmonologist, private | Rabat |
| Abdelkbir SAAF, MD | Pneumo-phthisiologist, private | Rabat |
| Abdellatif BOUSTA, MD | Pulmonologist & allergist, private | Marrakech |
| Abdelouahed MAHBOUB, MD | Pulmonologist & allergist, private | Casablanca |
| Abderrahim EL MAAMMAR, MD | Pulmonologist, private | Taza |
| Adil CHAIBAINOU, MD | Pulmonologist & allergist, private | Beni Mellal |
| Afaf THOUIL, MD | CHU Mohammed VI, Pulmonology service, Professor | Oujda |
| Ahmed EL OUTMANI, MD | Pulmonologist, private | Meknes |
| Arabi NACIRI, MD | Pneumo-phthisiologist, private | Casablanca |
| Aziz OUARSSANI, MD | Moulay Ismail Military Hospital, Pulmonology service | Meknes |
| Aziza GHANIM, MD | CHU Ibn Sina, Pneumo-phthisiologist | Rabat |
| Azzeddine MOHAMMADI, MD | Pulmonologist & allergist, private | Marrakech |
| Bouchra AMARA, MD | CHU Hassan II, Pulmonology service, Professor | Fes |
| Charkaoui CHERKI, MD | Pulmonologist, private | El Jadida |
| El Hassan ASSBAAI, MD | Duc de Toval Hospital, CDTMR | Tangier |
| Fatima KARKOS, MD | Pulmonologist, private | Ait Melloul |
| Fatima Zahra MAHBOUB, MD | Pulmonologist & allergist, private | Casablanca |
| Fatimazzahra SQALLI HOUSSAINI, MD | Clinic Al Hamd, Pulmonologist & allergist | Temara |
| Ferdaous SAHNON, MD | Pulmonologist & allergist, private | Tetouan |
| Hakima FEKKAK, MD | Pneumo-phthisiologist, private | Khouribga |
| Hatim KOUISMI, MD | CHU Mohammed VI, Pulmonology service | Oujda |
| Hicham NAJI AMRANI, MD | Oued Eddahab Military Hospital, Pulmonology service | Agadir |
| Hind SERHANE, MD | CHU Souss Massa, Pulmonology service, Professor | Agadir |
| Jamal Eddine BOURKADI, MD | Moulay Youssef Hospital, Pulmonologist | Rabat |
| Jamal SEBTI HANAFI, MD | Pulmonologist & allergist, private | Casablanca |
| Kacem ZAOUAK, MD | Pulmonologist & allergist, private | Casablanca |
| Khadija BAZZI, MD | Pulmonologist, private | Taroudant |
| Khadija ELFADI, MD | Provincial hospital, Pneumo-phthisiologist | Fkih Ben Salah |
| Khalid TAZI SIDQI, MD | Pulmonologist, private | Casablanca |
| Laila Herrak, MD | CHU Ibn Sina, Pneumo-phthisiologist, Professor | Rabat |
| Lamya CHRIF MORAND, MD | Pulmonologist & allergist, private | Kenitra |
| Lamyae AMRO, MD | Arrazi Hospital, Pulmonology service, Professor | Marrakech |
| Leila MALIKI, MD | Pulmonologist, private | Marrakech |
| Maria BERRADA, MD | Pulmonologist & allergist, private | Casablanca |
| Mariam RACHIDI, MD | Pulmonologist, private | Marrakech |
| Merbouha ABDERRAQUIB, MD | Pulmonologist, private | Khouribga |
| Mhamed EL MOUSSATI, MD | Pneumo-phthisiologist & allergist, private | Tetouan |
| Mohammed AHRAM, MD | Pneumo-phthisiologist & allergist, private | Sidi Kacem |
| Mohammed BOUAYAD, MD | Pulmonologist & allergist, private | Fes |
| Mohamed Chakib BENJELLOUN, MD | CHU Hassan II, Pulmonology service, Professor | Fes |
| Mohammed EL BIAZE, MD | CHU Hassan II, Pulmonology service, Professor | Fes |
| Mohamed EL KHOMSI, MD | Pulmonologist, private | Meknes |
| Mohamed OUTALEB, MD | Pneumo-phthisiologist, private | Agadir |
| Mohamed RACHID, MD | Pulmonologist, private | Marrakech |
| Mohamed RAGRAGUI, MD | Pulmonologist, private | Agadir |
| Mohamed STOULI, MD | Pulmonologist & allergist, private | Meknes |
| Mounia SERRAJ, MD | CHU Hassan II, Pulmonology service, Professor | Fes |
| Mountasser BENTHAMI, MD | Pulmonologist & allergist, private | Nador |
| Mustapha EL FTOUH, MD | CHU Ibn Sina, Pneumo-phthisiologist, Professor | Rabat |
| Naaima ZEMED, MD | Pulmonologist & allergist, private | Temara |
| Nabil EL MASBAHI, MD | Pulmonologist, private | Safi |
| Nadia AMANGAR, MD | Pulmonologist & allergist, private | Rabat |
| Noreddine JMILI, MD | Pulmonologist, private | Rabat |
| Saadia EL HASSANI, MD | Pulmonologist, private | Inzegane |
| Safae OUKILI, MD | CHU Tangier, Pneumo-phthisiologist resident | Tangier |
| Salim NACIRI, MD | Pneumo-phthisiologist, private | Sale |
| Samira EL HORD, MD | Pulmonologist & allergist, private | Fes |
| Samira MOKAHLI, MD | Pulmonologist & allergist, private | Oujda |
| Sanaa HAMMI, MD | CHU Tangier, Pneumo-pathologist, Professor | Tangier |
| Youssef EL ALAOUI BENCHAD, MD | Pulmonologist & allergist, private | Agadir |
